# An Integrated Genomic, Proteomic, and Immunopeptidomic Approach to Discover Treatment-Induced Neoantigens

**DOI:** 10.3389/fimmu.2021.662443

**Published:** 2021-04-15

**Authors:** Niclas Olsson, Marlene L. Heberling, Lichao Zhang, Suchit Jhunjhunwala, Qui T. Phung, Sarah Lin, Veronica G. Anania, Jennie R. Lill, Joshua E. Elias

**Affiliations:** ^1^ Department of Chemical and Systems Biology, Stanford School of Medicine, Stanford University, Stanford, CA, United States; ^2^ Mass Spectrometry Platform, Chan Zuckerberg Biohub, Stanford, CA, United States; ^3^ Department of Microchemistry, Proteomics and Lipidomics, Genentech, South San Francisco, CA, United States; ^4^ Department of OMNI Biomarker Development, Genentech, South San Francisco, CA, United States

**Keywords:** antigen presentation, IFN-γ, MHC, cancer, HLA, mass spectrometry, ligandome, neoantigen

## Abstract

All nucleated mammalian cells express major histocompatibility complex (MHC) proteins that present peptides on cell surfaces for immune surveillance. These MHC-presented peptides (pMHC) are necessary for directing T-cell responses against cells harboring non-self antigens derived from pathogens or from somatic mutations. Alterations in tumor-specific antigen repertoires — particularly novel MHC presentation of mutation-bearing peptides (neoantigens) — can be potent targets of anti-tumor immune responses. Here we employed an integrated genomic and proteomic antigen discovery strategy aimed at measuring how interferon gamma (IFN-γ) alters antigen presentation, using a human lymphoma cell line, GRANTA-519. IFN-γ treatment resulted in 126 differentially expressed proteins (2% of all quantified proteins), which included components of antigen presentation machinery and interferon signaling pathways, and MHC molecules themselves. In addition, several proteasome subunits were found to be modulated, consistent with previous reports of immunoproteasome induction by IFN-γ exposure. This finding suggests that a modest proteomic response to IFN-γ could create larger alteration to cells’ antigen/epitope repertoires. Accordingly, MHC immunoprecipitation followed by mass spectrometric analysis of eluted peptide repertoires revealed exclusive signatures of IFN-γ induction, with 951 unique peptides reproducibly presented by MHC-I and 582 presented by MHC-II. Furthermore, an additional set of pMHCs including several candidate neoantigens, distinguished control and the IFN-γ samples by their altered relative abundances. Accordingly, we developed a classification system to distinguish peptides which are differentially presented due to altered expression from novel peptides resulting from changes in antigen processing. Taken together, these data demonstrate that IFN-γ can re-shape antigen repertoires by identity and by abundance. Extending this approach to models with greater clinical relevance could help develop strategies by which immunopeptide repertoires are intentionally reshaped to improve endogenous or vaccine-induced anti-tumor immune responses and potentially anti-viral immune responses.

## Introduction

Peptides presented on the cell surface by major histocompatibility complex proteins (MHC; also known as human leukocyte antigen (HLA) in humans) are a fundamental component of immunological diseases, including infection, autoimmunity, allergy, and cancer. Decades of seminal research established robust mechanisms for antigen presentation by MHC-I and MHC-II molecules (1–4). Due to their importance across the span of human disease, intense efforts have built pMHC prediction algorithms to suggest which of trillions of possible peptides could have therapeutic utility (5). However, empirical data generated by high-throughput mass spectrometry methods have increasingly suggested large numbers of naturally processed peptide ligands do not fit conventional models (6, 7). Potential reasons behind these discrepancies include altered cell states, proteasomal changes, post-translational modifications, and modulation by antigen presentation-associated aminopeptidases, which these models largely cannot consider.

Anticipating antigen presentation by MHC molecules has been particularly desirable in the context of cancer-specific mutations. Many neoplasms avoid immune detection by altering their antigen presentation machinery through loss of heterozygosity, and by modulating their responses to inflammation and cytokine signaling (8, 9). If nonsynonymous mutations occur in expressed proteins and are readily presented by MHC molecules, they could serve as highly specific antigens (neoantigens) against which potent T-cell based immunotherapies could be targeted. However, dynamic cancer cell states complicate neoantigen prediction. Although including parameters trained on multiple empirical factors can improve predictive models (10–14), they still focus on the intrinsic nature of a given antigen’s potential for being presented by a MHC molecule. A wide array of intracellular and extracellular variables influences immunopeptide repertoires by changing protein expression, modulating antigen presentation machinery, or both (6, 7, 11). Modeling cell state-dependent changes in antigen presentation machinery is difficult, but can be readily assessed by routine proteomic surveys.

Knowledge of the tumor proteome can inform how changes in cytokine signaling responses, antigen presentation machinery levels, and cellular proteolysis, for example, can influence how a tumor’s immunopeptide repertoire is distinct from the surrounding healthy tissue. Regulated cellular proteolysis in particular is known to play a major role in MHC Class I-peptide repertoire generation. Inflammatory cytokines such as IFN-γ induce major shifts in antigen presentation machinery and activate T cell responses through enhancement of phagocytosis. IFN-γ directly stimulates the expression of several interferon-stimulated genes (ISGs) (15) having antibacterial, antiviral and anticancer (16) activities. Furthermore, IFN-γ leads to several constitutive catalytic proteasome subunits being replaced by the inducible subunits PSMB9 (LMP2) and PSMB8 (LMP7), respectively. The resulting “immunoproteasome” has been demonstrated to increase the abundance and diversity of MHC class-I ligands (16–18). In addition, prolonged IFN-γ exposure inevitably leads to increased availability of cytokine-induced translation products for MHC-mediated presentation, and to differentially expressed antigen presentation machinery such as ERAP proteins (19). Consequently, if such responses were pharmaceutically induced *in vivo*, they could alter global ligandome profiles or change the abundances of specific neoantigens in ways that could promote therapeutically beneficial immune responses. Numerous studies have examined IFN-γ’s transcriptome level effects (20), but to our knowledge, few in-depth combined proteogenomic and ligandome discovery profiling studies have been reported (21).

We propose that analyzing changes in immunopeptidome repertoires in the context of underlying protein expression changes can give mechanistic contexts to innumerable disease-specific candidates one might predict from DNA sequencing data (22). To test this, we performed whole-exome sequencing and in-depth mass spectrometric profiling of both the proteomes and the MHC-I and -II antigen repertoires of a human mantle cell lymphoma cell line (GRANTA-519) with and without exposure to IFN-γ. We mapped clear IFN-γ-dependent changes measured from the proteome to the corresponding immunopeptidome. Importantly, a substantial number of immunopeptidome changes had no apparent correlate at the protein level, and therefore are likely to be best explained by altered antigen presentation mechanisms. These data demonstrate that an exogenous cytokine like IFN-γ can qualitatively and quantitatively reshape antigen repertoires beyond what would be expected from expression changes alone. Furthermore, we identified antigens containing single nucleotide polymorphisms (SNPs; or neoantigens) on MHC-I and MHC-II only from IFN-γ-treated cells. This suggests that upregulated ISGs and changes in antigen presentation machinery could be viable modes for enhancing neoantigen presentation. This study demonstrates that a combined genomic, proteomic, and immunopeptidomic approach is a powerful way to discover novel presented antigens and tumor associated antigens, particularly when exogenous perturbations are applied. We believe this information will be valuable for advancing *in silico* prediction algorithms and therefore in developing and optimizing personalized and T-cell based immunotherapies that take into consideration a patient’s specific cytokine environment or drug treatment history.

## Materials and Methods

### Cell Culture

We profiled the GRANTA-519 cell line that was originally established from the peripheral blood at relapse of a high-grade B-NHL (leukemic transformation of mantle cell lymphoma, stage IV) diagnosed in a 58-year-old woman (https://web.expasy.org/cellosaurus/CVCL_1818). GRANTA-519 cells were maintained in RPMI 1640 medium with L-glutamine (HyClone™ GE Healthcare Life Sciences, Logan, Utah) with the addition of 10% fetal bovine serum (BenchMark Gemini Bio-Products, Sacramento, CA) and 1x of Penicillin-Streptomycin-L-Glutamine (Corning Mediatech Inc., Manassas, VA). Triplicate cell cultures were exposed to IFN-γ (carrier free; R&D Systems Inc., Minneapolis, MN) at a dosage of 10 ng/ml (initially reconstituted in sterile water to 0.2mg/mL and then diluted in RPMI 1640 media) for 24 hours. Following the IFN-γ 24-hour treatment the cells were harvested and washed twice with ice-cold PBS, snap-frozen in liquid nitrogen and then stored at -80°C until further sample preparation.

### Exome Sequencing of the GRANTA-519 Cell Line

Whole exome-sequencing was performed on GRANTA-519 cells to generate a cell line-specific sequence database that could be used for proteome and ligandome MS-analysis. In brief, paired-end sequencing was performed using an Illumina HiSeq 2000 in a 2 x 75 bp format resulting in a total of 87,463,304 read pairs sequenced. Of these, 84,774,734 were assigned a quality score of 23 or higher (HTSeqGenie v4.0.1) and were subsequently mapped to the reference GRCh38 genome (GSNAP ver. 2013-11-01 with arguments –M2 –n 10 –B 2 -i 1 –pairmax-dna=1000 –terminal-threshold=1000 –gmap-mod=none –clip-overlap). Of these, 84,708,444 could be aligned, and 72,494,993 aligned uniquely and used for further analysis.

### RNA Sequencing of the GRANTA-519 Cell Line

RNA sequencing was performed on the GRANTA-519 cell line in a similar fashion to exome sequencing. Paired-end sequencing was performed using an Illumina HiSeq 2000 in a 2 x 75 bp format resulting in a total of 94,278,646 read pairs sequenced. Of these, 88,247,894 were assigned a quality score of 23 or higher (HTSeqGenie v4.0.1). Removing 2,269,277 rRNA contamination reads yielded 85,978,617 post-processed reads which were mapped to the reference GRCh38 genome (GSNAP ver. 2013-11-01 with arguments –M2 –n 10 –B 2 -i 1 –N 1 –w 200000 –E 1 –pairmax-rna=200000 –clip-overlap). Of these, 85,780,285 could be aligned, and 72,589,199 aligned uniquely and used for further analysis.

### Variant Calling

Coding sequence variants were called using VariantTools (v1.9.4) with default parameters. In total 853,170 transcript variants were identified, 53,533 of which bore non-synonymous coding variants (missense, frameshifts, stop gains and in-frame indels), corresponding to 14,980 genomic variants. Further, 19,905 transcript variants were also identified from RNA-Seq data with a lenient threshold of at least one read containing the variant. Transcript variants identified from RNAseq were translated *in-silico* to make variant proteins, without haplotype phasing. These were then added to the sequence variants deduced from exome sequencing, as described above. Thus, the fasta sequences acting as the search space consisted of protein sequences containing non-phased variants (19,905 variant protein sequences) plus all wild-type proteins from the RefSeq database (71,867 wild type proteins). Amino acid changes due to the variants were inferred using Variant Effect Predictor (VEP) (23). Variant protein sequences were constructed by replacing the wild type amino acids with the variant amino acids, as inferred by VEP. The 19,905 variants were selected such that any amino acid change was identifiable by VEP. Variants for which an amino acid change could not be inferred were not included among these 19,905 variants.

### HLA Typing

HLA typing was performed by KASHI Transplant (Portland, Oregon) by amplifying genomic DNA with HLA locus-based typing and/or sequence specific primers and probes. The major HLA-I alleles were determined to be HLA-A*02:01, HLA-A*02:05, HLA-B*07:02, HLA-B*50:01, HLA-C*06:02 and HLA-C*07:02. For HLA-II the alleles were determined to be HLA-DRB1*11:03, HLA-DRB1*15:01, HLA-DRB3*02:02, HLA-DRB5*01:01, HLA-DPA1*04:01, HLA-DPB1*04:01 and HLA-DQA1*03:01, HLA-DQB1*06:02.

### Total Proteome Sample Preparation, TMT Labeling and High-pH Reversed Phase Fractionation for Proteome Analysis

1 × 10^7^ cells from each control and IFN-γ treated culture were used for protein extractions. In brief, cells were lysed in 8M urea, 150 mM NaCl, 5 mM DTT, 50 mM Tris pH 8 supplemented with Complete Protease Inhibitor Cocktail tablet (Roche) and 1x Halt™ Protease and Phosphatase Inhibitor Cocktail (ThermoFisher Scientific). Resulting lysates were centrifuged at 13,200 rpm for 15 min. at room temperature. Supernatants were transferred to fresh test tubes and underwent a second round of centrifugation. The resulting clarified supernatant was reduced with 5 mM DTT for 30 min at 37°C, then alkylated with 14 mM iodoacetamide for 45 minutes at room temperature in the dark and then quenched with 5 mM DTT for 20 min at room temperature. Proteins were further isolated by methanol-chloroform precipitation, and the protein pellet was washed twice with acetone before being resuspended in 300 µl of 8M urea, 50 mM Tris pH 8. Total protein concentrations were determined using the Pierce™ BCA Protein Assay Kit (Pierce, Rockford, IL) before being diluted to 1 M urea using 50 mM Tris pH= 8 prior to digestion with Trypsin/Lys-C Mix (Promega, Madison, WI) at a ratio of 1:25 enzyme: substrate (16 hours at 37°C). The reaction was quenched with formic acid and the peptides desalted using a Sep-Pak C18 1 cc Vac Cartridge, 50 mg (Waters, Milford, MA). Labeling with tandem mass tag (TMT) reagents (Thermo Fisher Scientific) was performed as previously described (24). In brief, the following six TMT-tags were used from a ten-plex kit: TMT-126, TMT-127N, TMT-128C, TMT-129N, TMT-130C and TMT-131. Each TMT reagent (0.8mg per vial) was reconstituted in 40 μl of acetonitrile and incubated with corresponding peptide sample for 1 hr. The reaction was quenched with a final concentration of 0.3% (v/v) hydroxylamine for 15 min at room temperature. Samples were acidified with 25% formic acid to pH ~ 2. In order to assess the labeling efficiency, we combined 5 μl (10%) from each sample, desalted by StageTip (25) and then analyzed by LC/MS as described below. Guided by these results, each individual labeled sample was then mixed to deliver equal signal across all reporter ion channels. The peptide mixture was then desalted using a Sep-Pak C18 1 cc Vac Cartridge, 50 mg (Waters) and resuspended in 10 mM ammonium formate, pH 10. Peptides were then fractionated by high-pH reverse phase fractionation (26, 27) using a 65 min + 15 min step-gradient (buffer A (10 mM ammonium formate, pH 10) and buffer B (10 mM ammonium formate, 90% ACN, 10% H_2_O, pH 10) using an Agilent 1200 HPLC (Agilent Technologies, Santa Clara, USA). In total 84 fractions were collected, concatenated and combined (27) into a total of 12 fractions. These were then dried by vacuum centrifugation and using a C18 based StageTip, dried down and stored at -80°C until final LC-MS/MS measurement.

### Mass Spectrometry Analysis of the GRANTA-519 Proteome

Each peptide fraction was resuspended in 20 µl 0.1% formic acid, of which 10% of the material was injected. Peptides were separated on a 20 cm reverse-phase column (100 µm inner diameter, packed in-house with ReproSil-Pur C18-AQ 3.0 m resin (Dr. Maisch GmbH) over 160 min using a two-step linear gradient with 4–25% buffer B (0.2% (v/v) formic acid, 5% DMSO, and 94.8% (v/v) acetonitrile) for 120 min followed by 25-40% buffer B for 30 min at a 400 nl/min flowrate on an Eksigent Ekspert nanoLC-425 system (Sciex, Framingham, USA). The LC system was coupled on-line with an Orbitrap Elite instrument (Thermo Fischer Scientific, Bremen, Germany) *via* a nano-electrospray source. For quantification runs we used a profiling method applying the MultiNotch MS3 approach (28, 29). MS1 precursor ion scans were performed in the Orbitrap over the mass range of 400-1300 m/z, and the resolution of 60,000. The ten most intense ions (intensity above 500 counts) were selected for CID fragmentation and detection in the ion trap with precursor ion isolation width of 1.2 m/z, maximum ion time of 150 ms and normalized collision energy of 35% at activation time of 10 ms. Following each MS2 analysis, the 5 most intense fragment ions (intensity above 500 counts) were selected for HCD MS3 fragmentation with isolation width of 2.5 m/z, normalized collision energy of 50% at activation time of 2 ms. MS3 fragment ions were measured in the Orbitrap at a resolution of 30,000. The AGC settings were set to 1E6, 5E4 and 5E3 for FTMS1, FT MSn and IT MSn scans, respectively. Charge state screening was disabled to allow fragment ions to be selected for MS3. Dynamic exclusion was enabled with a repeat count of 1 with the repeat duration set to 55 seconds.

### Computational Interpretation of the GRANTA-519 Proteome

Raw data analysis was performed using ProteomeDiscover v2.1.0.81 (Thermo Fischer Scientific, San Jose, CA). For SEQUEST-HT searches the parent mass error tolerance was set to 20 ppm and the fragment mass error tolerance to 0.6 Da. Strict trypsin specificity was required allowing up to two missed cleavages. Carbamidomethylation of cysteine was set as fixed modification and oxidation of methionines as a variable modification. The minimum required peptide length was set to seven amino acids. Spectra were queried against a target-decoy sequence database consisting of the Uniprot human database (Aug-2015), GRANTA-519 cell line-specific sequences from the exome sequencing results and common contaminants and reversed versions of all the above sequences. A false discovery rate of 0.01 was required at both the peptide level and protein level. Known false positives (i.e. decoys) and contaminants were removed. Peptide identifications with observed signals in at least four of the six labeled channels were included for final statistical analysis. In order to identify differentially expressed proteins between the two groups of cells (control vs IFN-γ treated) the quantitative data was log-transformed followed by a t-test using Qlucore Omics Explorer v3.2 (Qlucore AB, Lund, Sweden). The criteria for being reported as differentially expressed were p<0.01 and a log2 fold-change of ≥1.2. These criteria were empirically selected to exclude proteins without clear relevance to the IFN-γ treatment, as inferred from enriched gene ontologies (see below). These criteria resulted in q-values < 0.197 when correction for multiple hypothesis testing using Benjamini-Hochberg (30) was applied. Pathway analysis of differentially expressed proteins identified in the proteome was performed by using QIAGEN’s Ingenuity^®^ Pathway Analysis (IPA^®^, QIAGEN Redwood City, www.qiagen.com/ingenuity).

### Purification of MHC-I and MHC-II Presented Peptides

In order to assess the ligandome sampling depth, pMHC-I and pMHC-II immunopeptidomes were extracted from different amounts of GRANTA-519 cells using 1x10^6^, 1x10^7^, 1x10^8^ and 1x10^9^ cells. Furthermore, to address the effects of IFN-γ, three biological replicate samples of IFN-γ treated vs untreated cells (a total of 5x10^8^ cells per replicate) were prepared. The MHC class-I or MHC class-II molecules were isolated and the associated peptides extracted as previously described (31–35) with some modifications. In brief, cells were lysed for 20 min on ice in 20 mM Tris-HCl (pH8), 150 mM NaCl, 1% (w/v) CHAPS, 0.2 mM PMSF, 1x Halt™ Protease and Phosphatase Inhibitor Cocktail (ThermoFisher Scientific) supplemented with Complete Protease Inhibitor Cocktail (Roche, Mannheim, Germany). The lysate was centrifuged (2x30 min, 13,200 rpm at 4°C) and the resulting supernatant was precleared for 30 min using rProtein A Sepharose fast-flow beads (GE Healthcare, Uppsala, Sweden). For all HLA-1 immunoprecipitations, the precleared lysate was incubated with the pan HLA-A-, B-, and C- antibody W6/32 (36) coupled to rProtein A Sepharose fast-flow beads for 5h at 4°. Following the immune-capture of HLA-1, the supernatant was transferred to new tubes, precleared for 1 hour using rProtein A Sepharose fast-flow beads (GE Healthcare, Uppsala, Sweden) and then incubated with the HLA-DR specific antibody L243 (37) (produced and purified by Genentech from hybridoma) coupled to rProtein A Sepharose fast-flow beads overnight at 4°C. Following the serial immune-captures of MHC-I and MHC-II, the beads were washed with TBS (pH 7.4) and peptides were eluted from the purified MHC molecules using 10% acetic acid. Peptides eluted from both immunocaptures were passed through a 10 kDa MWCO size filter, followed by a concentration step using vacuum centrifugation before being desalted on C18-based StageTips (25) and stored at -80°C until LC-MS/MS analysis.

### AQUA Peptide Quality Control

In total 30 synthetic peptides covering a total of 22 SNP sites and two potential neoantigens, were purchased from CPC Scientific (Sunnyvale, CA) with a purity of >70%, dissolved in 0.1% FA and quality checked at a concentration of 250 fmol by mass spectrometry. One pool of 25 MHC-I peptides (a total of 23 SNP peptides covering 19 sites and two neoantigens) and one pool of 5 MHC-II peptides (covering 3 SNP sites) were then spiked into the purified immunopeptidome preparations from the GRANTA-519 cell line cultures together with retention time standard peptides (Pierce, Rockford, IL).

### Mass Spectrometry Analysis of Immunopeptidomes

For MS analysis of the HLA ligandomes, isolated peptides were reconstituted in 12 µl 0.1% FA and analyzed on an LTQ Orbitrap Elite mass spectrometer (Thermo Fischer Scientific, Bremen, Germany) as previously described (34). Samples were separated by capillary reverse phase chromatography on a 20 cm reversed phase column (100 µm inner diameter, packed in-house with ReproSil-Pur C18-AQ 3.0 m resin (Dr. Maisch GmbH)) over a total run time of 160 min using a two-step linear gradient with 4–25% buffer B (0.2% (v/v) formic acid, 5% DMSO, and 94.8% (v/v) acetonitrile) for 120 min followed by 25-40% buffer B for 30 min using the Eksigent Ekspert nanoLC-425 system (Sciex, Framingham, USA). Three injections were made per sample with different instrument methods: higher energy collisional dissociation (HCD) and collision induced dissociation (CID) including single-charged species, and CID excluding single-charged species. Acquisition was executed in data dependent mode with the full MS scans acquired in the Orbitrap mass analyzer with a resolution of 60000 and m/z scan range 340-1600. The top ten most intense ions with masses ranging from 700-1800 Da for all pMHC-I samples and from 700-2750 Da for pMHC-II samples were then selected for fragmentation and the fragmented ions were analyzed in the Orbitrap mass analyzer at a resolution of 15,000 (FWHM). The ions were fragmented with a normalized collision energy of 35% and an activation time of 5 ms for CID and 30 ms for HCD. Dynamic exclusion was enabled with repeat count of 2, repeat duration of 30 s and exclusion duration of 30s. The minimal signal threshold was set to 500 counts. Furthermore, for samples containing AQUA peptides, a precursor mass inclusion list was utilized in the collision induced dissociation (CID) including single-charged species.

### Computational Identification and Quantification of Immunopeptides From Mass Spectra

All tandem mass spectra were queried against a “target-decoy” sequence database (38) consisting of the human proteome Uniprot database (Aug-2015), GRANTA-519 cell-specific sequences from the exome sequencing results and common contaminants and reversed versions of all the above sequences. All spectra were searched using both SEQUEST (39) and PEAKS DB search engines (40). Spectra were also interpreted by *de novo* sequencing (PEAKS Studio 7, Bioinformatics Solutions Inc.) to improve high-confidence peptide identification. The parent mass error tolerance was set to +/-10 ppm and the fragment mass error tolerance to 0.02 Da. Enzyme specificity was set to none and oxidation (M), deamidation (N,Q), cysteinylation (C), and phosphorylation (S, T, Y) were considered as variable modifications. High-confidence peptide identifications were selected at a 1% false discovery rate with the Percolator algorithm (41). We optimized Percolator’s input for proteogenomic immunopeptide analysis (35). These modifications include denoting whether the *de novo* sequence matches database assignments; the magnitude of scores assigned by all three search algorithms; the source of the identified peptide (Swiss-Prot, TrEMBL, or our own sequencing); and the presence of one or more post-translational modifications in the identified peptide. Unlike conventional proteome analysis, false discovery rates were not evaluated at the level of assembled proteins, as this would unnecessarily penalize proteins identified by just one peptide. Quantitative abundance values (MS1 peak areas) were calculated as previously described (42). Skyline (43) and Xcaliber (Thermo Fisher Scientific, San Jose, CA) were used to determine the absolute abundance of the AQUA-peptides.

### Evaluation of Core Epitopes and Predicted Affinity Values

Immunopeptidome datasets were evaluated with the PLAtEAU script (44) to identify core binding epitopes. Only peptides reported in all three biological replicates (with the criteria of at least observed in one of the three technical replicate injections) were considered for further PLAtEAU analysis. The minimum epitope length was set to 9 for the MHC-I and both 9 and 13 for the MHC-II. The default option of impute with lowest measured value in each run was enabled. Both the entire set of peptides from the ligandome analysis and the subsequently defined core epitope output from PLAtEAU were used as input for affinity prediction using NetMHCpan 4.1 (45) for MHC-I data and NetMHCIIpan 4.0 (10) for the MHC-II data set. The predicted rank threshold for strong binding peptides were 0.500 or a rank threshold for weak binding peptides of 2.000 for MHC-I and a rank threshold of 10 for MHC-II were included for analysis. The highest affinity allele across the six GRANTA-519 alleles which met the above criteria was considered for further analysis.

### Analysis of Immunopeptide Gene Ontologies With Respect to Underlying Proteome

A computational strategy was developed to assess changes at the immunopeptidome level upon treatment with IFN-γ with respect to changes in the underlying proteome. This uses as input the abundance rank of each peptide in the control state and IFN-γ state as well as the rank of 126 differentially expressed proteins at the proteome level. Peptides were included in the analysis if they were found in at least 2 biological replicates, and the mean abundance was used for final input. Peptides that were uniquely presented in the IFN-γ state that could not be explained by changes in the underlying proteome were subject to GO Term analysis using The Database for Annotation, Visualization and Integrated Discovery (DAVID) v6.8 (46, 47).

## Results

### Rationale and Experiment Design

**Figure 1:**
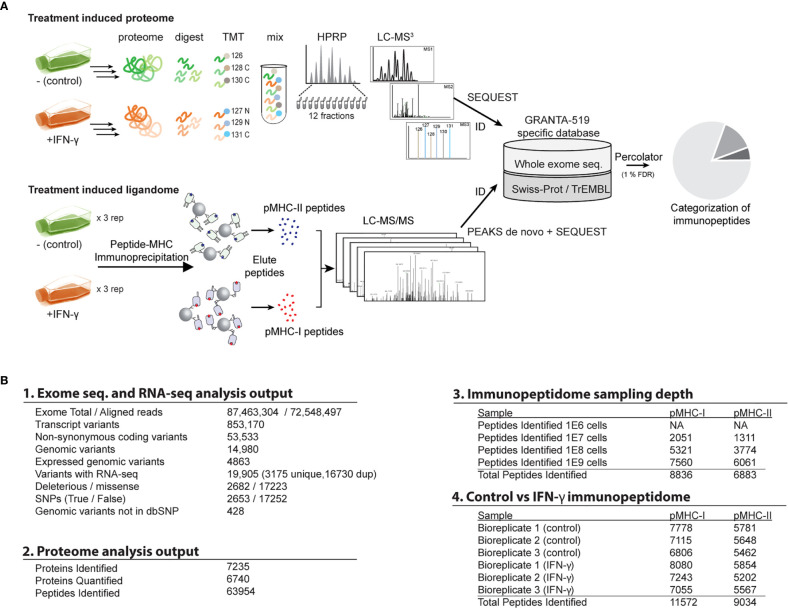
Overview of the study workflow for whole exome sequencing, proteome, and immunopeptidome analyses. **(A)** Schematic of the study design and experimental workflow. See main text for additional details. **(B)** Summary of output from 1. exome and RNA-seq, 2. proteome analysis, 3. Cell input vs immunopeptidome sampling depth evaluation, 4. immunopeptidome of control vs IFN-γ treated samples.

Our experimental approach (**Figure 1**) combines genomic, proteomic and immunopeptidomic analyses to discover IFN-γ -induced pMHC and neoantigens. To better understand how IFN-γ alters pMHC repertoires, we developed a categorization scheme which distinguishes pMHC resulting from differential expression versus from differential presentation.

### Exome and RNA Sequencing Uncovers Potential Neoantigens From GRANTA-519 Cell Line

Obtaining specimen-matched, deep genomic sequencing is an essential prerequisite for most neoantigen discovery efforts. We performed exome sequencing of the GRANTA-519 cell line. We identified 4,863 high-confidence non-synonymous sequence deviations from the GRCh38 reference genome (**Figure 1B**). Of these, 4,435 genomic variants were already described in dbSNP (48), leaving 428 novel mutation candidates. Although an untransformed parental genome was not available for this cell line, we estimate that 8.8% of these non-synonymous sequence variants could be cancer-specific, as they bear no similarity to any SNPs catalogued within the COSMIC database (49). However, for the purpose of this report, we consider both putative SNPs and mutations as candidate neoantigen sources (**Figure 1**).

### Interferon Gamma Affects Global Protein Expression and Antigen Processing Machinery

**Figure 2:**
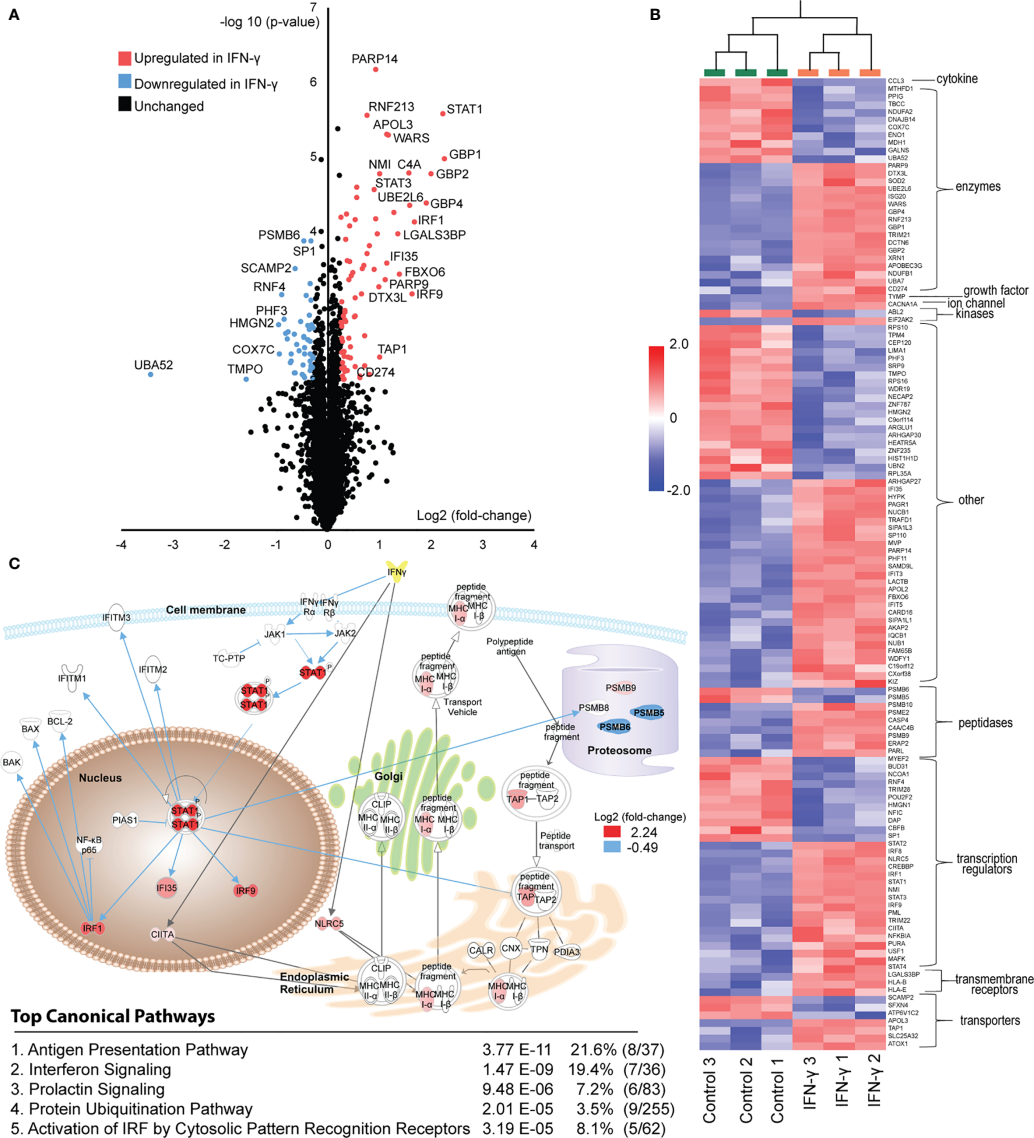
Induced proteome changes in GRANTA-519 after 24 hours of IFN-γ exposure. **(A)** Volcano-plot for all the 6,740 quantified proteins over the three biological replicate experiments. In order to identify differentially expressed proteins between the IFN-γ and controls a t-test (p-value <0.01, q-value < 0.196 and a fold-change of 1.2) was applied. **(B)** Hierarchical clustering was performed (using the agglomerative clustering algorithm) and grouped based on molecular functions (from IPA-mapping). In total 126 differentially expressed proteins were identified. The clustered data was color-visualized using standard z-score over all variables for each sample. **(C)** Biological relevance of differentially expressed proteins between controls and IFN-γ treated cells determined using IPA. The 126 proteins identified as significantly differentially expressed proteins between the control and interferon gamma treated proteomes were analyzed in Ingenuity Pathway analysis software using core-analysis. The top five canonical pathways are listed. The schematic graph includes proteins from the antigen presentation pathway and the interferon signaling pathway. The gray arrows are linked to proteins within the antigen presentation pathway and blue arrows associated with the interferon signaling. Log2-ratios were imported into IPA; red denotes up-regulation, and blue denotes down-regulation in the interferon-gamma treated state. Upstream regulator analysis of these differentially expressed proteins with Ingenuity Pathway Analysis (IPA) predicted IFN-γ -dependent activation with a z-score of 6.375 (p-value 3.3e-23).

IFN-γ induces the expression of about 200 interferon-stimulated genes (ISGs) (15, 20, 50, 51) which could promote novel antigen presentation in two ways: most obviously, newly expressed ISGs are available for novel antigen presentation. Alternatively, IFN-γ could expand antigen repertoires by regulating antigen presentation machinery (16, 18). Under both possibilities, entirely new protein sources could be made available for presentation by MHC molecules. However, where the former might be predicted from conventional expression analyses, the latter possibility may require empirical pMHC measurements. To distinguish these two possibilities for individual proteins, we first surveyed the GRANTA-519 global proteome with and without IFN-γ stimulation. Our analysis yielded 63,954 unique peptides derived from 7,235 proteins (1% FDR at both peptide and protein levels) (**Figure 1B.2**). Of 6,740 proteins that were quantified with high confidence (**Supplemental Table 1**, **Supplemental Figure 1** ), just 126 (1.94%) surpassed fairly lax criteria for differential expression (**Figure 2A**).

Nevertheless, known interferon signaling, and antigen presentation pathway components were significantly enriched among 78 proteins upregulated by IFN-γ (**Figures 2B, C** **)**. These include key STAT-family transcription regulators (STAT1 and STAT3) and interferon regulatory factor family members (IRF1, IRF8 and IRF9). Furthermore, 43 differentially expressed proteins were reported to be direct IFN-γ targets (**Figure 2C**). These include several members of the guanylate-binding protein family (GBP1, GBP2 and GBP4) which mediate potent antiviral and antimicrobial responses while restraining inflammation-induced cellular proliferation (52). In addition, programmed cell death 1 ligand 1 (PDL1/CD274) demonstrated a 1.55 fold increase following IFN-γ exposure (p < 0.0089) (**Figure 2A**). PD-L1, a widely publicized immunotherapy target (53), has previously been reported to be regulated by JAK-STAT signaling pathways downstream of IFN-γ receptor activation (54, 55). While these findings support our cell culture model and proteomic profiling measurements, they point to specific ISGs we might expect to be differentially presented upon IFN-γ stimulation.

In addition to expected interferon-pathway components, we identified that several essential antigen presentation pathway components were differentially expressed. These include proteasome subunits that were either down regulated (PSMB5 and PSMB6) or upregulated (PSMB9 and PSMB10) (**Figure 2C**), consistent with previous reports of immunoproteasome induction by IFN-γ (16, 17). We further observed an upregulation of ERAP2, the caspase inhibitor CARD16, and TAP1 (**Figure 2A**), all of which have well-characterized roles in refining antigen presentation by HLA-I (19, 56); their differential expression is therefore expected to alter MHC Class I immunopeptidome repertoires. Of note, our exome sequencing data indicates that GRANTA-519 cells possess the K392N variant of ERAP2 (fasta file available at www.ebi.ac.uk/pride/archive under accession number PXD020750). This is one of two major alleles of this gene within the population (19, 57), yet is not expressed under most circumstances due to nonsense-mediated mRNA decay. We further observed a 1.55 fold increase in HLA-B expression (p<0.009) increase following IFN-γ exposure, (**Figure 2B**), which is expected to have an overall effect on the presented immunopeptidome pool by increasing presentation of peptides specifically with affinity for the HLA-B alleles. The effect of IFNs on HLA-B is well established (58–61). In terms of proteins related to the class II machinery, we observed an upregulation of the MHC class II transactivator (CIITA) with a 1.33 fold increase following IFN-γ exposure, (**Figure 2B**). CIITA is essential for transcriptional activity of the HLA class II promoter. These data collectively indicate that in our model system, a short IFN-γ exposure caused a modest but identifiable change in protein expression levels of key molecules within both the interferon regulated network and the antigen presentation machinery. These proteome-level changes led us to suspect that IFN-γ could induce much larger changes to the MHC-presented ligand repertoires, potentially enhancing tumor-associated antigen and neoantigen presentation. Based on these data, we hypothesized substantial changes to the MHC-I immunopeptidome – such as increased diversity of unique MHC-I ligands – could occur.

### Establishing an Immunopeptidome Workflow for Detecting SNPs and Neoantigens

We and others demonstrated that rare somatic mutation-bearing cancer neoantigens can be presented by MHC and measured by mass spectrometry (34, 35, 62–64). However, we subsequently showed that this type of neoantigen was difficult to detect, particularly in cancers with low-mutation burden. Thus, detecting IFN-γ-induced SNPs and neoantigens could hinge on the pMHC assay’s sensitivity. Hence, prior to measuring the effect of IFN-γ stimulation on the GRANTA-519 immunopeptidomes, we set out to evaluate the performance of our profiling platform by performing a single preparation of MHC-I and MHC-II ligandome analyses with a range of 1 x 10^6^ – 1 x 10^9^ GRANTA-519 cells as input (**Figure 1B**). The MHC-I and –II ligandomes we measured with these experiments adhered to expected length distributions (**Supplemental Figure 2A**). Furthermore, 99% of all unique pMHC we identified were restricted to each corresponding MHC-I (8,615 out of 8,836) or MHC-II (6,662 out of 6,883) isolation experiment (**Supplemental Figure 2B**) and not identified in both. Both observations support the validity and specificity of our overall immunopeptidome methodology. Of more than 15,000 unique pMHC-I and pMHC-II identified, just 40 (20 MHC-I and 20 MHC-II) contained a SNP which was exclusively attributed to exome sequencing (**Supplemental Figure 2C**). No overlap among SNP peptides or the associated SNP source proteins between the MHC-I presented and the MHC-II presented peptides was observed. The number of identified SNP-containing peptides ranged from 3-18 per analysis for MHC-I, and 1-18 for MHC-II (**Supplemental Figure 2C**). Within this dilution series of cell input we identified one putative neoantigen (ALGPPPFGL) from tapasin isoform 1 TAPBP (not a previously known SNP); presented by MHC-I in the 1 x 10^9^ cell preparation. Based on the acquired immunopeptidome sequence depth we decided that 5 x 10^8^ cells per replicate would give a reasonable depth for the triplicate biological replicate immunopeptidome measurements with and without IFN-γ stimulation.

### Interferon Gamma Stimulation Reshapes MHC-I and MHC-II Immunopeptidomes

Triplicate biological replicate measurements were performed to isolate ligands presented by MHC-I and MHC-II with and without IFN-γ stimulation (**Figure 1**). The replicates demonstrated a high degree of analytical consistency, with 49% of pMHC-I and 45% of pMHC-II consistently identified among biological replicates (**Figure 1B** and **Supplemental Figure 3A**). In order to increase our confidence in defining an IFN-γ-induced immunopeptidome from GRANTA-519 cells, we restricted the majority of our subsequent analysis to the pMHC that were identified in all three biological replicates. When applying this strict filter, we found 951 unique pMHC-I that were only identified and presented in all three replicates in the IFN-γ state and were never identified in the control-treated state **(** **Supplemental Figure 3B** **).** Conversely, 733 unique pMHC-I were identified from all three control state experiments, and never from IFN-γ state. Unique pMHC-II repertoires were reproducibly observed in the control or IFN-γ states in the same manner as for pMHC-I. For example, 582 unique pMHC-II were identified and presented in all three replicates in the IFN-γ state and not in the control state (**Supplemental Figure 3B**). The majority of pMHC-I were found to be distributed fairly evenly among the four HLA-A and HLA-B alleles while the pMHC-II repertoire was dominated by those predicted to bind a single locus and allele (HLA-DRB1*11:03). Of note, we found a clear distinction in HLA preference among pMHC-I exclusively identified following IFN-γ treatment: just 36.3% were predicted to bind HLA-A while over half (52.2%) were predicted to bind HLA-B. This contrasts with the pMHC-I exclusively identified in the control state, in which over half (51.3%) were predicted to bind HLA-A while 41.4% were predicted to bind HLA-B. These values are significantly different from the proportion of all pMHC-I expected to bind either locus (41.7% HLA-A; 49.5% HLA-B; p=2.5e-5 (control); p=5.4e-4 (IFN-γ); chi-squared test) (**Supplemental Figure 3C**). Similar studies have shown larger increases in the HLA-B specific immunopeptidomes in response to IFN-γ (61, 65). The more modest difference in HLA-B response we observed here may be due to shorter incubation times as well as lower doses of IFN-γ. Additionally, both previous studies were performed in epithelial cells, whereas this study was performed in a lymphoma cancer cell line which may have higher basal levels of IFN-γ signaling.

### Interpreting MHC-I and MHC-II Presented Core Epitopes

**Figure 3:**
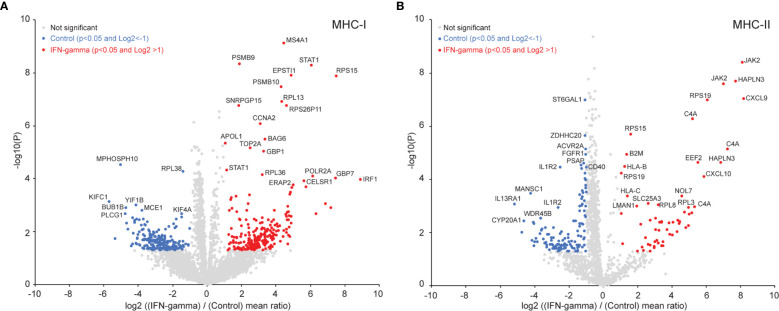
Induced immunopeptidome epitope changes in GRANTA-519 after 24 hours of IFN-γ exposure. **(A)** Volcano-plot for all the MHC-I core epitopes quantified by peptide landscape antigenic epitope alignment utility (PLAtEAU) (44). The number of differentially presented epitopes between the IFN-γ and controls (with a p-value <0.05 and a log2 fold-change of >1) resulted in 236 core epitopes which were increased in the IFN-γ state (red) and 201 that were higher in control state (blue data points). Adjusting for multiple hypothesis testing (p<0.05, Benjamini-Hochberg) resulted in 44 core epitopes that were increased in the IFN-γ state and 8 that were increased in the control state. A selected set of these core epitopes are labeled with their protein names in the plot. **(B)** Volcano-plot for all the MHC-II core epitopes quantified by PLAtEAU. Sixty core epitopes were increased in the IFN-γ state, and 126 were increased in the control (non-Benjamini-Hochberg corrected p-value <0.05; log2 fold-change >1). Adjusting for multiple hypothesis testing (Benjamini-Hochberg) resulted in 45 core epitopes which were increased in the IFN-γ state and 65 that were increased in the control state. A selected set of these core epitopes are labeled with their protein names in the plot.

**Figure 4:**
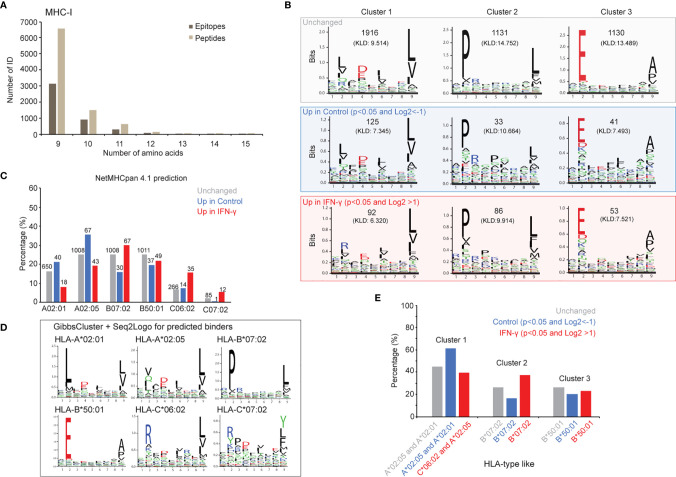
IFN-γ exposure does not uniformly affect antigen presentation across all alleles. B-allele presented core epitopes induced after 24 hours of IFN-γ exposure are supported by MHC binding predictions and ab initio motif discovery. **(A)** A total of 4,732 MHC-I core epitopes were inferred by PLAtEAU with a minimum epitope length of 9, and presence in all three biological replicate experiments. **(B)** Independent GibbsCluster analysis of all core epitopes. All core epitopes for the three cohorts (non-significant (grey box), increased in control state (blue box) and increased in the IFN-γ state (red box)) were analyzed with GibbsCluster (version 2.0) with MHC-I default parameters. The three top-reported clusters for each condition are presented and the Kullback-Leibler Distance (KLD) score is listed in brackets. **(C)** Evaluation of the predicted binding affinities for all observed core epitopes. Epitopes showing no significant abundance difference between IFN-γ and control states (“Unchanged”, grey); increased abundance in the control state (“Up in control”, blue); and increased abundance in the IFN-γ state (“Up in IFN-gamma”, red) were assigned to one of GRANTA-519’s six HLA-I alleles by NetMHCpan 4.1. Epitopes were scored as weak (rank binding score <2.000) or strong binders (rank binding score <0.500) per allele, and the lowest-scoring (predicted strongest binding) was plotted for each epitope-allele assignment. Cases of equal rank binding score values <2.000 between multiple alleles, were reported as a binder for all the corresponding alleles. **(D)** The largest core epitope group (“unchanged”) was used to determine sequence motifs specific to this data set. GibbsCluster was performed on epitopes predicted to bind each allele (NetMHC pan), and the resulting motifs were created with Seq2Logo. **(E)** The relative proportion of MHC I epitopes in clusters 1-3 in part **(B)** with their likely HLA-type are shown.

Given that different pMHC could span a single binding core in multiple length and register configurations, we sought to simplify our analysis by condensing multiple pMHC into single core antigenic sequence they have in common. Applying the recently developed PLAtEAU algorithm (44) to our immunopeptidome datasets enabled this simplification, and helped us consolidate overlapping pMHC intensities into individual binding core epitope representations. Considering only ligands that were both identified and quantified (9,790 pMHC-I and 8,489 pMHC-II; **Supplemental Table 2**), our analysis yielded 4,732 core epitopes extracted from pMHC-I (**Figure 3A**, **Figure 4A**, **Supplemental Table 3A**) and 1,337 core epitopes extracted from pMHC-II (**Figure 3B**, **Supplemental Figure 4**, **Supplemental Table 3B** **)**. We found 437 (9.4%) of pMHC-I core epitopes were differentially presented between the IFN-γ and control states (**Figure 3A**). Core epitopes from proteins like STAT1, ERAP2, IRF1 that increased with IFN-γ in our proteome data (**Figure 2**) also demonstrated increased MHC-I presentation (**Figure 3A**). In addition, several regions from PSMB9 and PSMB10 were among pMHC-I that increased in the IFN-γ state, further supporting immunoproteasome induction by interferon, and consistent with our proteomics data. Considering the core epitopes identified from pMHC-II, 186 (13.9%) were differentially presented between the IFN-γ state and the control state (**Figure 3B**). Interestingly, pMHC-II core epitopes derived from HLA-B, HLA-C, Beta-2-microglobulin (B2M) and JAK were among those which increased with IFN-γ. Given that the JAK/STAT is the major, but not the only, pathway linked to the IFN-γ response, it is not surprising we observed a significant increased presentation of HLA-B and two JAK derived epitopes on MHC-II.

We note that while most pMHC we identified were represented to similar extents with and without IFN-γ treatment, several appeared to be restricted to the control or IFN-γ states (**Supplemental Figure 3B**). To explore whether these differences corresponded with amino acid biases within the binding epitopes, we deduced MHC-I and MHC-II core epitopes with PLAtEAU and then extracted sequence motifs from each of these three categories (unchanged, enriched in control, enriched with IFN-γ treatment). We found multiple amino acid and positional restrictions from both pMHC-I (**Figure 4B**) and pMHC-II (**Supplemental Figure 4B**) repertoires, as determined by GibbsCluster analysis. While pMHC-I restrictions were largely consistent regardless of their IFN-γ specificity, they seemed far more variable among pMHC-II (**Supplemental Figure 4B**). To further refine these directly inferred motifs, we first assigned all epitopes to the GRANTA-519 cells’ MHC-I and MHC-II alleles with NetMHCpan (see *Materials and Methods*). We next produced empirically-derived motifs from the set of pMHC which did not change between control and IFN-γ states with GibbsCluster: each motif elucidation analysis was restricted to the set of core epitopes NetMHCpan assigned to each of GRANTA-519’s MHC-I (**Figures 4C, D** **)** and MHC-II **(** **Supplemental Figures 4C, D** **)** alleles.

We found the consensus binding motifs deduced from bulk pMHC assignments (**Figure 4B**) were particularly consistent with the known motifs extracted from each MHC-I allele (**Figure 4D**), whereas strong MHC-II motifs were less discernable (**Supplementary Figures 4B–D**). Despite their consistency in composition across the experimental conditions we tested, we note that pMHC-I motif frequencies were markedly different between those enriched in either control or IFN-γ states (**Figure 4B**). For example, pMHC-I epitopes with prolines at the second position (**Figure 4B**, Cluster 2), were depleted in the control versus the IFN-γ state (16.2% versus 36.9%; **Figure 4E**). This dominant proline motif is most similar to that deduced from epitopes assigned to the HLA-B*07:02 allele (**Figure 4D**). Conversely, motifs reminiscent of HLA-A*02 alleles (**Figure 4B**, Cluster 1) were enhanced in the control versus IFN-γ state (61.3% versus 39.5%; **Figure 4E**). We further note that sequences matching a HLA-C*06:02 motif, with arginine at the second position, likely contributed to this cluster, as supported by the direct NetMHC allele assignment (**Figure 4D**). Taken together, these data demonstrate that IFN-γ-dependent presentation is not uniform across all MHC-I alleles.

While we were unable to discern IFN-γ-dependent changes among MHC-II motifs, we note several IFN-γ-specific MHC-II core epitopes derived from unusually short 9-mer peptides that originated from ribosomal subunit proteins. By scoring the underlying 9-mer peptides against all GRANTA-519 cells’ MHC I and MHC II allele-specific binding motifs (66), we found most were predicted to bind HLA-A or HLA-B alleles more so than the HLA-DR alleles (**Supplemental Figure 5**). This led us to conclude that these peptides are likely contaminants from the MHC-I immunoprecipitation since MHC-I and MHC-II immunoprecipitations (*Materials and Methods*) were serially performed from the same cell lysates (67). We point out, however, that these 9-mers could nevertheless be of biological significance since these peptides were only found in the IFN-γ-treated MHC-II peptides and not in the control state (68).

### A Strategy for Interpreting MHC-Presented Antigens With Respect to the Underlying Proteome

**Figure 5:**
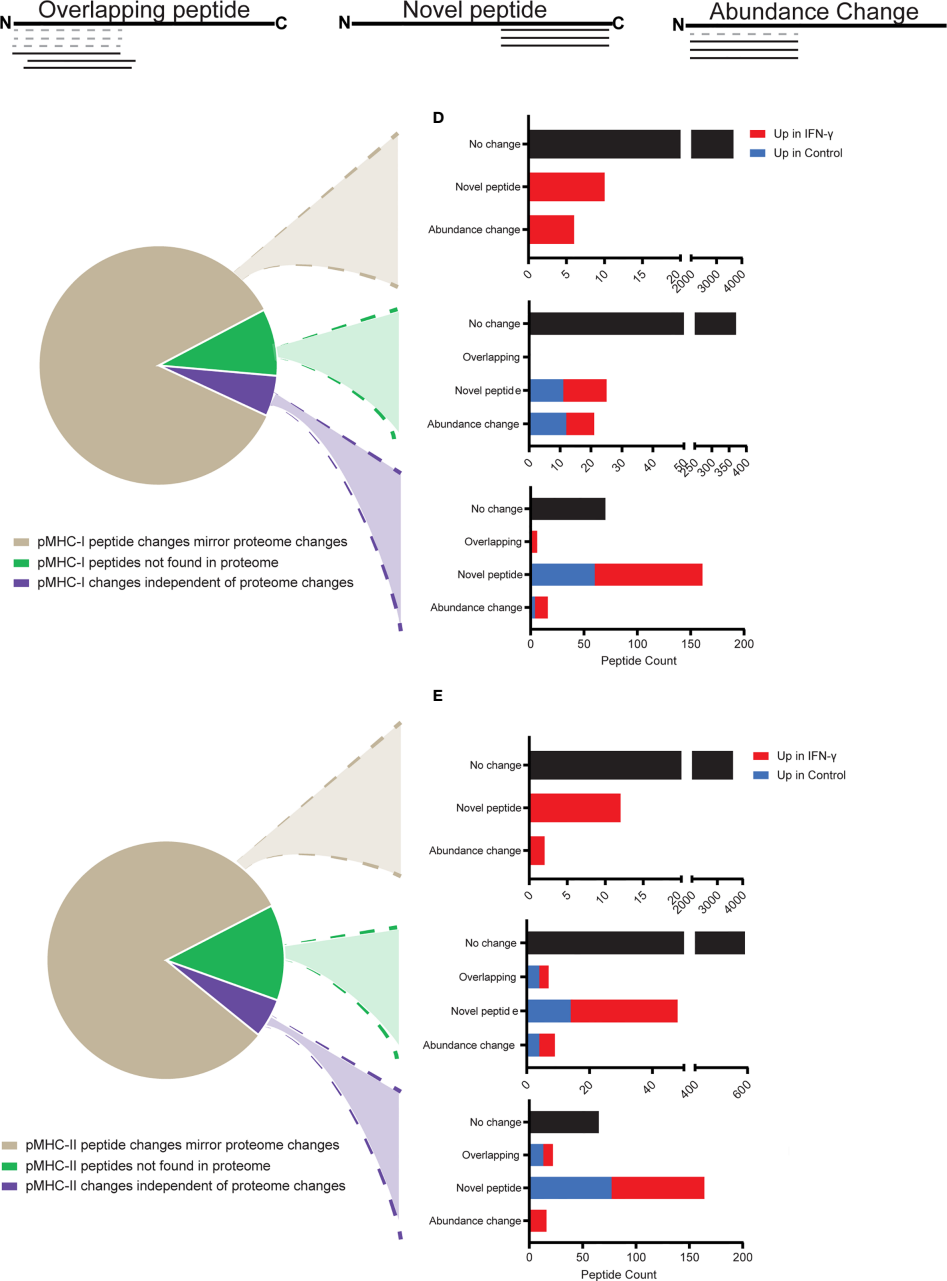
Interpreting MHC-presented antigens with respect to the underlying proteome. pMHC were categorized with respect to the manner in which their abundances and their underlying protein abundances changed between control and IFN-γ-treated samples. Only pMHCs identified with high-confidence, and which demonstrated low variance (fractional rank difference <0.2) across replicates were considered. **(A)** Schematic of three major peptide categories comparing peptides identified in the control state (dashed lines) or IFN-γ state (solid lines), both mapped onto the underlying protein’s primary amino acid sequence arranged from the N- to C-terminus. **(B, C)** pMHC-I **(B)** and pMHC-II **(C)** were classified in 3 categories: pMHC changed in abundance between control and IFN-γ states in a manner that mirrored changes measured at the proteome level (tan), pMHC which were derived from proteins not measured from the parallel proteome analysis (green), and pMHC demonstrating abundance profiles (changed or unchanged) that did not correspond with the abundance of the underlying protein (unchanged or changed) (purple). **(D, E)** pMHC-I **(D)** and -II **(E)** can be further categorized based on whether they showed no substantial abundance change between control and IFN-γ states (black), or were consistent with the three categories described in **(A)**.

To further understand the cells’ response to IFN-γ and effects at the immunopeptidome level, we developed a computational strategy to categorize pMHCs into discrete categories based on the how pMHC repertoires and corresponding proteomes changed following IFN-γ treatment (**Figure 5**). These categories involved qualitative and quantitative changes measured from the pMHC sequences we identified (**Figure 5A**) and by integrating data from both proteome and pMHC analyses (**Figures 5B–C**). We found that most (85% of pMHC-I, 81% of pMHC-II) pMHC mirrored their corresponding protein’s abundance across control and IFN-γ states (**Figures 5B, C**, tan wedge). As expected from our proteomic analysis (**Figure 2A**), the vast majority (>99.5%) of these pMHC showed no abundance change following IFN-γ treatment. However, we found 16 pMHC-I and 14 pMHC-II were presented in an IFN-γ-dependent manner (**Figures 5D, E**, top bar charts). These 16 pMHC-I derived from 10 proteins which demonstrated both induced protein expression and induced presentation. Not surprisingly, they originated from IFN response genes, including proteasome subunit beta type-10 (PSMB10), antigen peptide transporter 1 (TAP1), NF-kappa-B inhibitor alpha (NFKBIA), guanylate binding protein 4 (GBP4), E3 ubiquitin-protein ligase TRIM21, signal transducer and activator of transcription 1-alpha/beta (STAT1), E3 ubiquitin-protein ligase RNF213 (RNF213), poly [ADP-ribose] polymerase 14 (PARP14) and Protein NLRC5 (NLRC5) (**Supplemental Table 4**).

We found 16 pMHC-II derived from 5 proteins which changed in abundance consistent with the underlying protein measurements. Of note, eight of these pMHC-II were derived from Complement C4-A (C4A). Increased mRNA stability leading to increased C4A synthesis after IFN-γ stimulation has previously been reported (69).

We found substantial numbers of pMHC-I (9.1%) and pMHC-II (13.1%) that were derived from proteins that were not found in the proteome data set (**Figures 5B, C**, green wedge). While most (91.4%) of these pMHC did not change with IFN-γ exposure (**Figures 5D, E**, middle bar charts), 46 (pMHC-I) to 74 (pMHC-II) did, most of which were induced by IFN-γ. It is possible that the surprisingly large proportion of pMHC we identified that were not reflected in our proteome analysis could be attributed to class I and class II antigen presentation pathways to sample from low-abundant proteins. These proteins may have fallen below our detection threshold for proteomic identification, but may be disproportionately represented in the immunopeptidome. Relatedly, defective ribosome products (DRiPs) and other types of unstable proteins might not contribute to the measurable proteome, but would be expected to be well-represented in immunopeptidome repertoires.

Finally, we found that 6% of pMHC changed in a manner independent of the proteome (**Figures 5B, C**, purple wedge). Of these, most were novel peptides exclusively identified in the control or IFN-γ conditions. 253 pMHC-I in this category were derived from 208 proteins. Of these, 12 pMHC-I from 12 different proteins were present in both control and IFN-γ conditions but were substantially upregulated after IFN-γ stimulation. Further, 101 of these independent pMHC-I from 97 proteins were identified only in the IFN-γ treated samples. GO term analysis of the proteins from which these novel pMHC-I were derived indicated that they reflected the same biological pathways, cellular components, and molecular functions as the other pMHC-I described in this study (**Supplemental Figure 6A**). We found 87 pMHC-II exclusively in the IFN-γ state that did not correspond to changes in the underlying proteome. GO term analyses revealed no significant or substantial differences between these novel IFN-γ pMHC-II relative to all pMHC-II identified here (**Supplemental Figure 6B**). We note, however that 26 of these were 9-mer peptides and likely to be bound by contaminating MHC-I molecules (**Supplemental Figure 5**).

Two clear differences between the MHC-I and MHC-II immunopeptidomes were apparent from evaluating the categories noted in **Figure 5**, and accounting for the effects of IFN-γ treatment. First, the frequency of novel pMHC (as defined in **Figure 5A**) was higher in the MHC-I immunopeptidome (101 novel pMHC in the IFN-γ state, 60 novel pMHC in the control state) compared to only 61 novel pMHC-II in the IFN-γ state (excluding 9-mers) and 77 novel pMHC-II in the control state (**Figure 5**, **Supplemental Tables 4B, C**). Furthermore, while there were just 6 pMHC-I found in the IFN-γ state that overlapped with or were nested within pMHC-I found in the control state, there were 22 such pMHC-II (**Figure 5**, **Supplemental Tables 4B, C**). This may be due to HLA-DR’s ability to bind a broader range of peptides lengths compared to MHC-I alleles. In summary, there are more novel pMHC-I and more overlapping pMHC-II upon IFN-γ exposure. Ultimately, while these data demonstrate that most changes in pMHC repertoires might be predicted from the underlying proteome, a strategy like the one shown here could be useful for illuminating neoantigens that are presented under certain cell states. Such pMHC may be of particular interest if they would largely evade immune selection under steady-state conditions.

### Are SNPs Passengers Along for the Ride?

**Figure 6:**
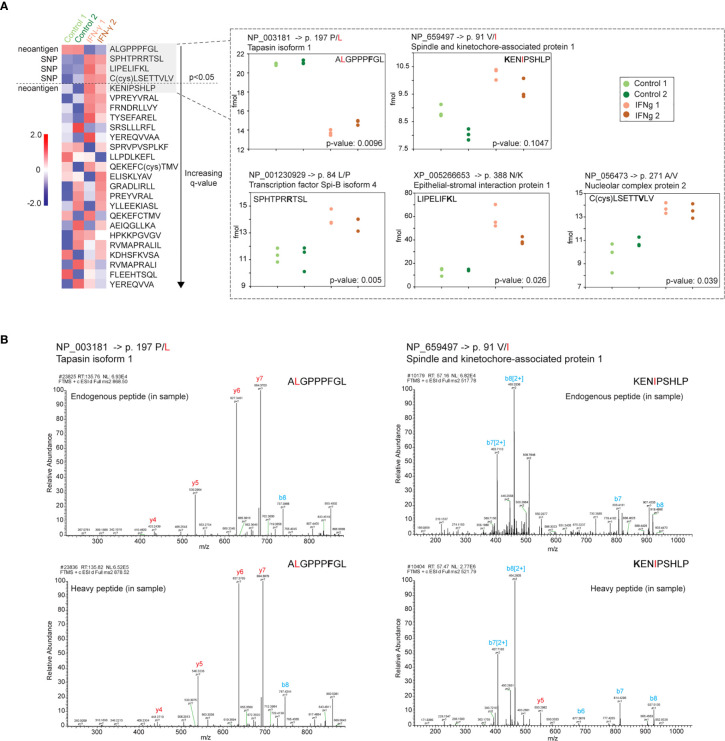
Validation and absolute quantification of pMHC-I bearing SNPs and two neoantigens. Isotopically labeled synthetic peptides were used to evaluate the abundance of two endogenous neoantigens and 19 SNP sites presented on MHC-I. **(A)** Four of 25 SNP and neoantigen pMHCs demonstrated differential abundance (p-value <0.05 and q-value <0.49). One of these four peptides was a potential neoantigen originated from tapasin isoform 1 (ALGPPP**F**GL) and had a proline to leucine amino acid change at position 2 (amino acid variants indicated in red; isotopically labeled amino acid in standard peptide indicated in bold). The second neoantigen peptide originated from a spindle and kinetochore-associated protein (**K**ENIPSHLP) with a valine to isoleucine substitution but no significant abundance difference between control and IFN-γ states (p-value 0.1047). Heatmap: The mean for three technical replicate injections per biological replicate (n=2 biological replicates) was used in a t-test between the control state and interferon-gamma state. Heatmap depicts a standard z-score calculated from the four averaged values (two control treatment means, green) versus two IFN- γ means, orange) per pMHC. pMHC are sorted by q-values (Benjamini-Hochberg corrected). Peptide-specific abundance plots: endogenous peptide quantified based on abundance measured relative to isotopically labeled standard spiked into specimen (see *Materials and Methods*). **(B)** MS/MS spectrum of both the endogenous and heavy synthetic AQUA peptides for the two neoantigen peptides.

One potential benefit of the approach described above is the ability to discover treatment-specific pMHCs bearing tumor neoantigens. In order to more precisely evaluate potential neoantigen presentation induced by IFN-γ, we synthesized 25 isotopically labeled peptides corresponding with two putative neoantigens and spanning 19 SNP sites identified from our initial pMHC-I analysis (**Figure 1B** **)**. Following an initial validation by LC-MS (data not shown), these heavy labeled peptides were spiked into the purified ligandome samples for two of the biological replicates we collected (**Figure 1A**). The majority of the targeted SNP sites demonstrated no differential expression. However, three endogenous pMHC-I SNP peptides demonstrated significant differential presentation after IFN-γ treatment (**Figure 6**). Furthermore, one neoantigen peptide ALGPPPGL from tapasin isoform 1 was identified to be significantly down-regulated after the IFN-γ treatment (**Figure 6A**) while the second neoantigen peptide was not found to be statistically differentially presented. MS/MS spectrum validation supported the identities of two neoantigen peptides and the three differentially presented pMHC-1 SNP-peptides. (**Figure 6B** and **Supplemental Figure 7**). However, the infrequency of SNP- and mutation-containing peptides among both unstimulated and stimulated cells potentially suggests their large-scale discovery by current mass spectrometry methods still may be difficult to achieve.

## Discussion

Here we showed that combining transcriptomics, proteomics, and immunopeptidomics can be a powerful strategy for uncovering treatment-specific effects on antigen presentation. Specifically, using an IFN-γ-treated cancer B cell line, we demonstrate a classification strategy designed to provide focus to pMHC presentation that would not be predicted based on expression data alone. We found that this category was more apparent among pMHC-I than pMHC-II, although it was substantially represented in both. We attribute this difference to the induction of the immunoproteasome by IFN-γ and the changes in expression of various components of MHC-I the antigen presentation machinery. These data support our recommendation that comparative immunopeptidomics experiments be accompanied by matched proteome analysis in order to uncover treatment effects that alter antigen presentation pathways without having a substantial impact on global transcription or translation.

Our analyses of pMHC following IFN-γ treatment revealed 101 pMHC-I that could not be predicted by changes at the proteome level. These peptides were derived from proteins that were not significantly different from most other proteins contributing to the pMHC-I repertoire in terms of GO term categories or peptide length, but NetMHC analysis and core epitope analysis using PLAtEAU revealed a sizeable distinction in B-allele presented core epitopes after 24 hours of IFN-γ exposure between control and treatment (**Figure 4**). Furthermore, the consensus binding motifs of putative MHC-I (and to a lesser-extent, MHC-II) alleles generated motifs that matched known ones.

The impact of IFN-γ on cancer cells is complex and being heavily investigated. Taken together, we show here that a low-dose IFN-γ treatment can lead to increased numbers of unique pMHC I and II, and that these novel pMHC are not strongly biased towards any particular subset of the proteome. Therefore, IFN-γ exposure shows promise as a way to improve outcomes of immunotherapy in a variety of clinical contexts. While this study indicates the potential therapeutic use of IFN-γ, it should be noted that systemic dosing with IFN-γ could upregulate the presentation of antigens in healthy tissue as well. Therefore, further studies in a more complex model system are necessary before IFN-γ can be considered for clinical use. We believe this approach can extend to innumerable other treatments, including commonly prescribe first-line interventions following an initial diagnosis.

## Data Availability Statement

The datasets presented in this study can be found in online repositories. The names of the repository/repositories and accession number(s) can be found below: https://www.ebi.ac.uk/pride/archive/, PXD020750 (70).

## Author Contributions

NO, JL, and JE designed the experiments. NO performed the experiments.VA provided the GRANTA-519 for pMHC dilution evaluation and performed pilot IFN-γ dosing evaluations. SJ performed exome sequencing, RNA sequencing, and subsequent variant calling analysis. JL and QP provided research reagents. NO, MH, LZ, SJ, QP and JE analyzed the data. SL wrote the peptide binning script with input from NO, MH, and JE. NO, MH, and JE wrote the manuscript with contributions from all authors, who commented on it at all stages. All authors contributed to the article and approved the submitted version.

## Funding

This work was supported by a Damon Runyon-Rachleff Innovation Award from the Damon Runyon Cancer Research Foundation (DRR-13-11; JE) the W.M. Keck Foundation Medical Research Program (JE), NIH NIDCR (grant R01-DE027750-01; JE) and the Chan Zuckerberg Biohub.

## Conflict of Interest

JL, VA, QP, and SJ are affiliated with Genentech. NO is affiliated with Calico Life Sciences LLC. LZ, SL and JE are affiliated with Chan Zuckerberg Biohub.

The remaining author declares that the research was conducted in the absence of any commercial or financial relationships that could be construed as a potential conflict of interest.
