# Supplementary material for: An Integrated Genomic, Proteomic, and Immunopeptidomic Approach to Discover Treatment-Induced Neoantigens
Source: Front Immunol. 2021 Apr 15;12:662443. doi: 10.3389/fimmu.2021.662443 (PMC8082494; doi:10.3389/fimmu.2021.662443)
Supplement: Supplementary file 1 [file DataSheet_1.pdf]

Supplementary Material

1 Supplementary Figures and Tables

1.1 Supplementary Figures

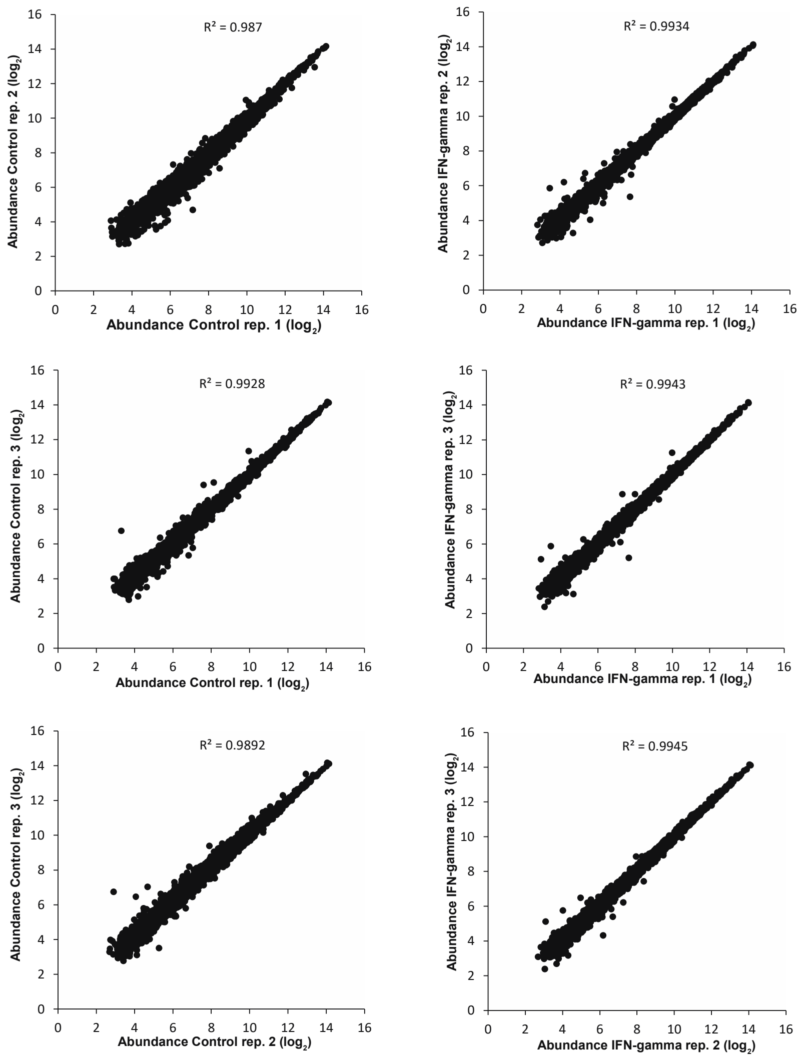

Supplemental Figure 1. Reproducibility of the proteomic analysis. Protein abundances measured from three biological replicates of untreated and IFN-γ treated cells were compared in a pairwise fashion. In total, 6,740 proteins were quantified (in four of the six samples), whereof 6727 were quantified in all six with pairwise high reproducibility (R^2-values between 0.98-0.99) for all experiments.

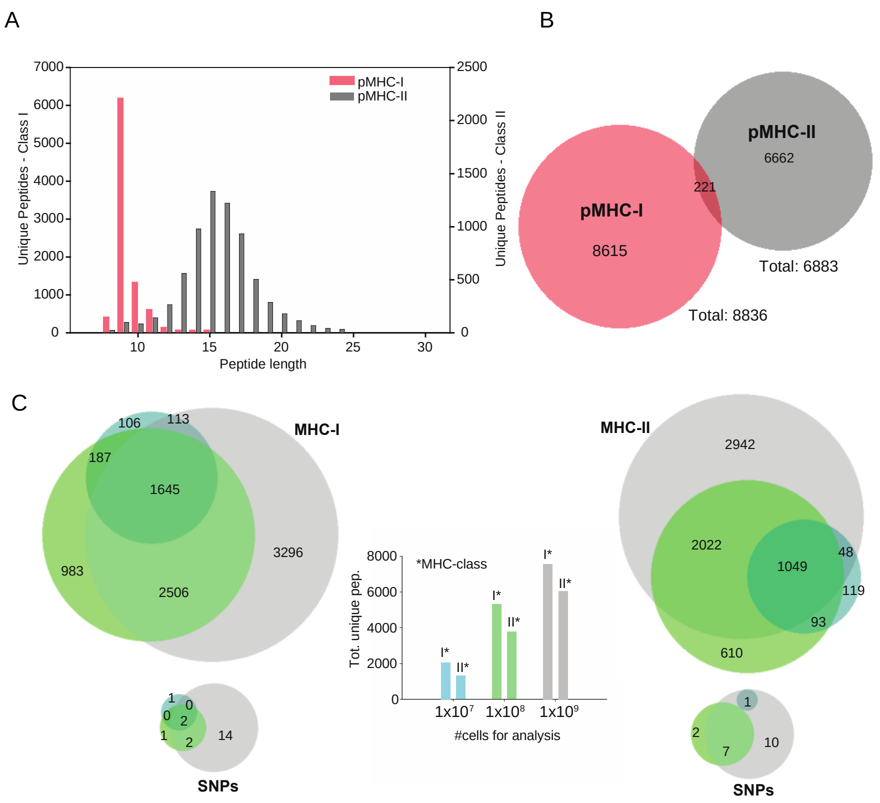

Supplemental Figure 2. Immunopeptidome sampling depth of pMHC-I and pMHC-II from GRANTA-519 cells. **(A)** Length distribution of pMHC-I and pMHC-II. **(B)** Venn analysis of all unique identified peptides from the pMHC-I vs pMHC-II dilution series experiments using cell inputs between 1 x 10^6 - 1 x 10^9 (Figure 1). **(C)** The effect of cell amount input on ligandome sampling depth. No peptides in the 1x10^6 condition passed the Percolator FDR filtering step. The number of SNP/neoantigen pMHC per cell amount are shown as separate Venn diagrams. Two observed SNPs (neoantigen) peptides with a potential conversion from isoleucine to leucine were excluded in the above statistics for pMHC-I due to limitation in differentiating isoleucine and leucine by the mass spectrometer.

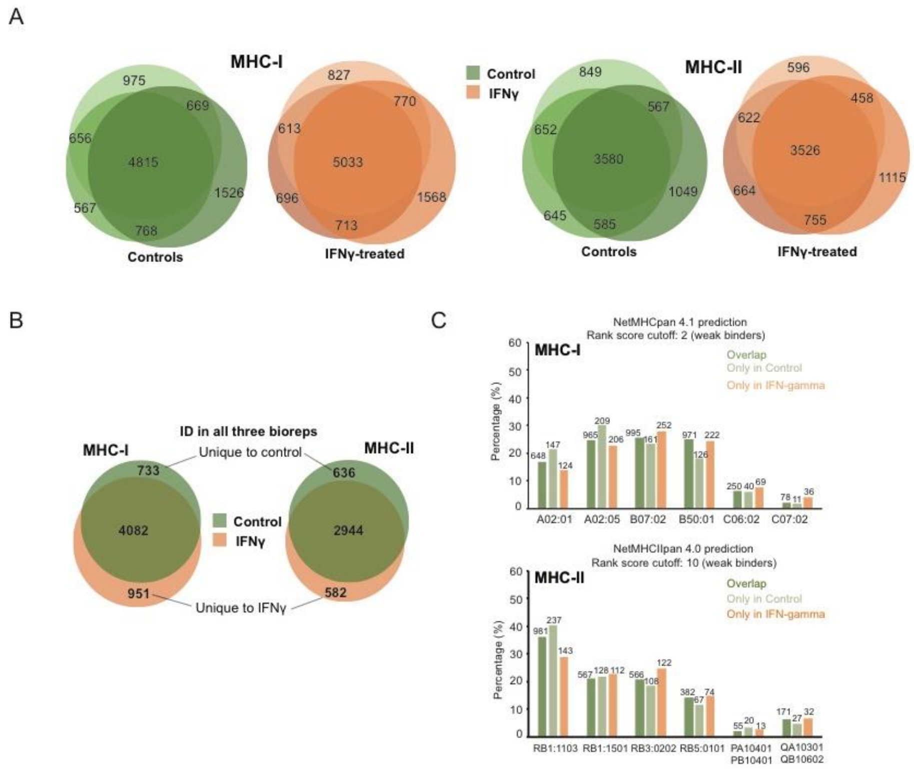

Supplemental Figure 3. IFN-γ induced immunopeptidomes. **(A)** Venn analysis of all unique pMHC identified across three biological replicates. **(B)** For all pMHC identified in all three biological replicates, Venn diagrams show the number of unique pMHC that were identified in control or IFN-γ states. **(C)** Evaluating pMHC binding predictions to GRANTA-519 HLA alleles. Total numbers of pMHC scored at least as weak binders per allele was determined by using NetMHC. Analyses were limited to peptides identified in all three replicates. The best scoring peptide across the six alleles with a rank threshold of 2 or lower for MHC-I or in the case of MHC-II a rank threshold of 10 or lower was kept and considered a binder. Hence, the best ranked allele for each peptide was used. In cases of equal rank between several alleles the given pMHC was reported for all the binding alleles. The percentage of peptides presented per allele was then determined by evaluating the total numbers of peptides scored as weak or strong binders per allele.

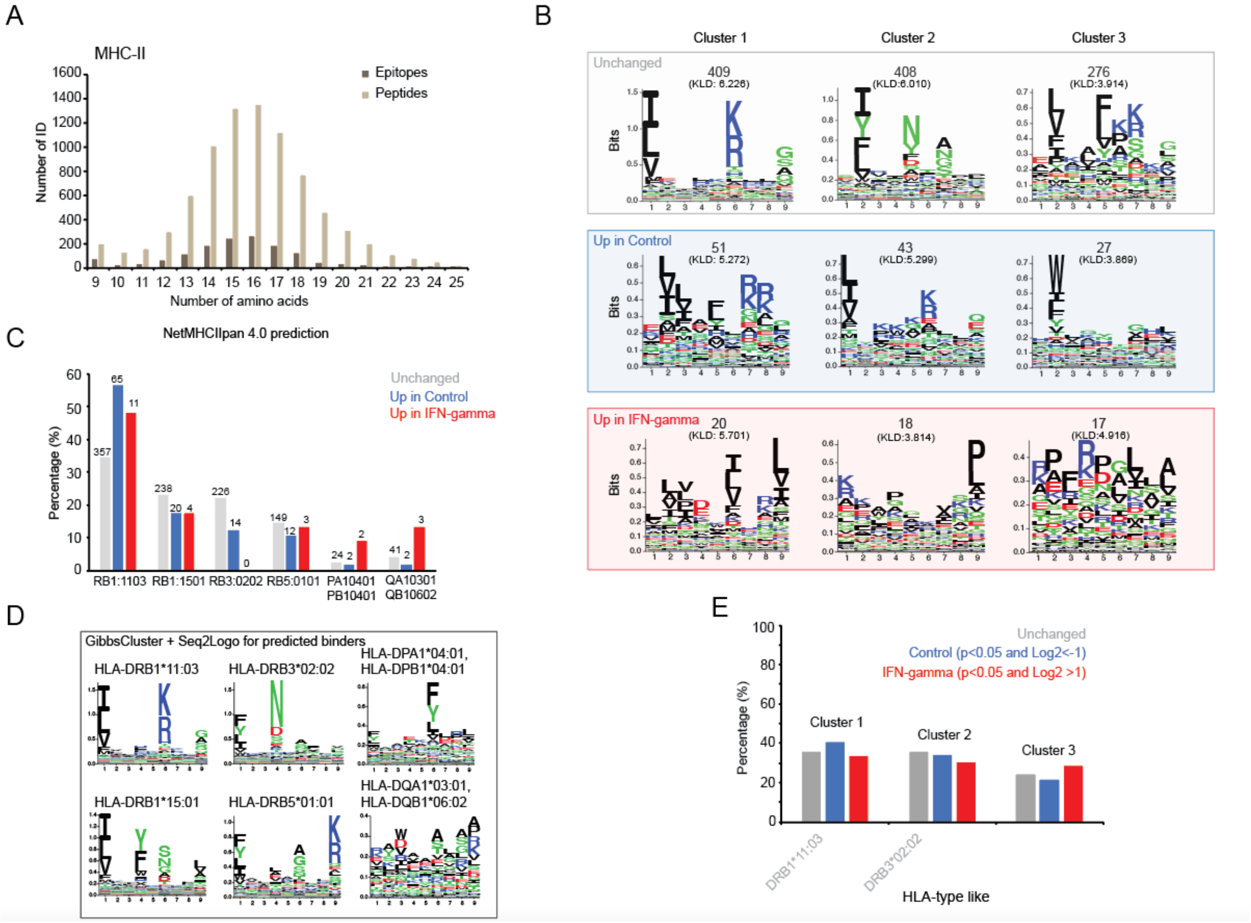

Supplemental Figure 4. Evaluation of core epitopes presented by MHC-II. (**A)** A total of 1337 core epitopes over the three biological replicate experiments were reported when using 9 amino acids as minimum epitope length with the by peptide landscape antigenic epitope alignment utility script(43). (**B**) Independent GibbsCluster analysis of all core epitopes. All core epitopes for the three cohorts (non-significant (grey box), increased in control state (blue box) and increased in the IFN-γ state (red box)) were analyzed with GibbsCluster (version 2.0) with MHC-II default parameters. The three top-reported clusters for each condition are presented and the Kullback-Leibler Distance (KLD) score is listed in brackets. (**C)** Evaluation of the predicted binding affinities for all observed core epitopes. Epitopes showing no significant abundance difference between IFN-γ and control states ("Not significant", grey); increased abundance in the control state ("Up in control", blue); and increased abundance in the IFN-γ state ("Up in IFN-gamma", red) were assigned to one of GRANTA-519's six HLA-II alleles by NetMHCpan 4.0. Epitopes were scored using the rank binding score cutoff of <10 per allele, and the lowest-scoring (predicted strongest binding) for each epitope-allele assignment was plotted. In cases of equal rank binding score values between multiple alleles, it was reported as a binder for all the corresponding alleles. (**D**) The largest group (non-significant epitopes) additional GibbsCluster was performed for each allele to generate motifs using Seq2Logo. (**E)** The relative proportion of MHC II epitopes in clusters 1-3 in part (B) with clear likely HLA-type are shown.

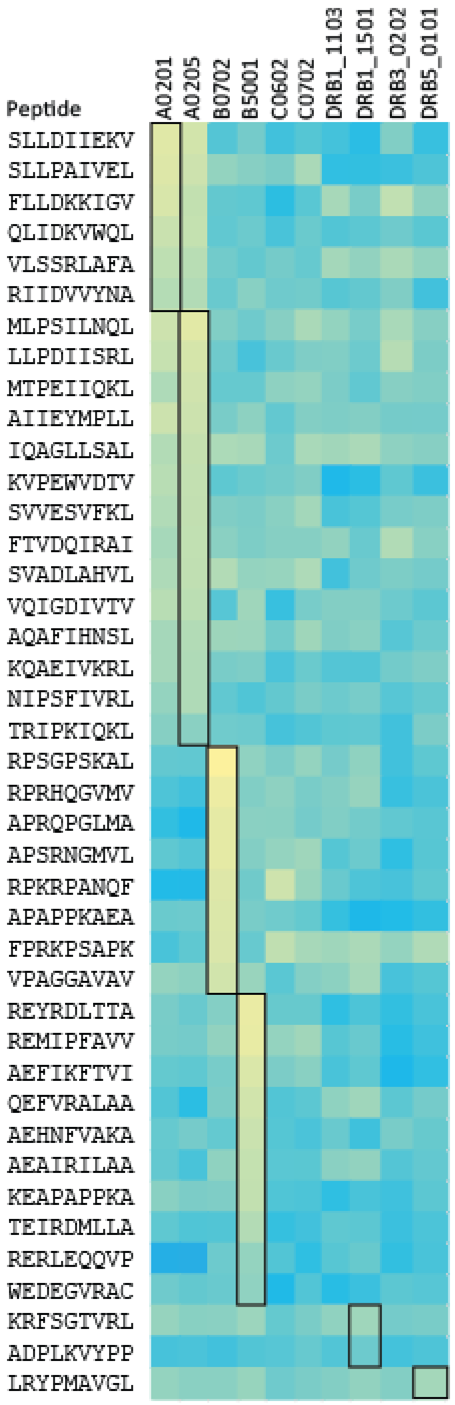

Supplemental Figure 5. 9-mer peptides eluted during the serial MHC-II immunoprecipitation following IFN-γ treatment. Peptides tend to favor HLA-A and HLA-B binding motifs. Black boxes indicate highest scoring allele for each peptide.

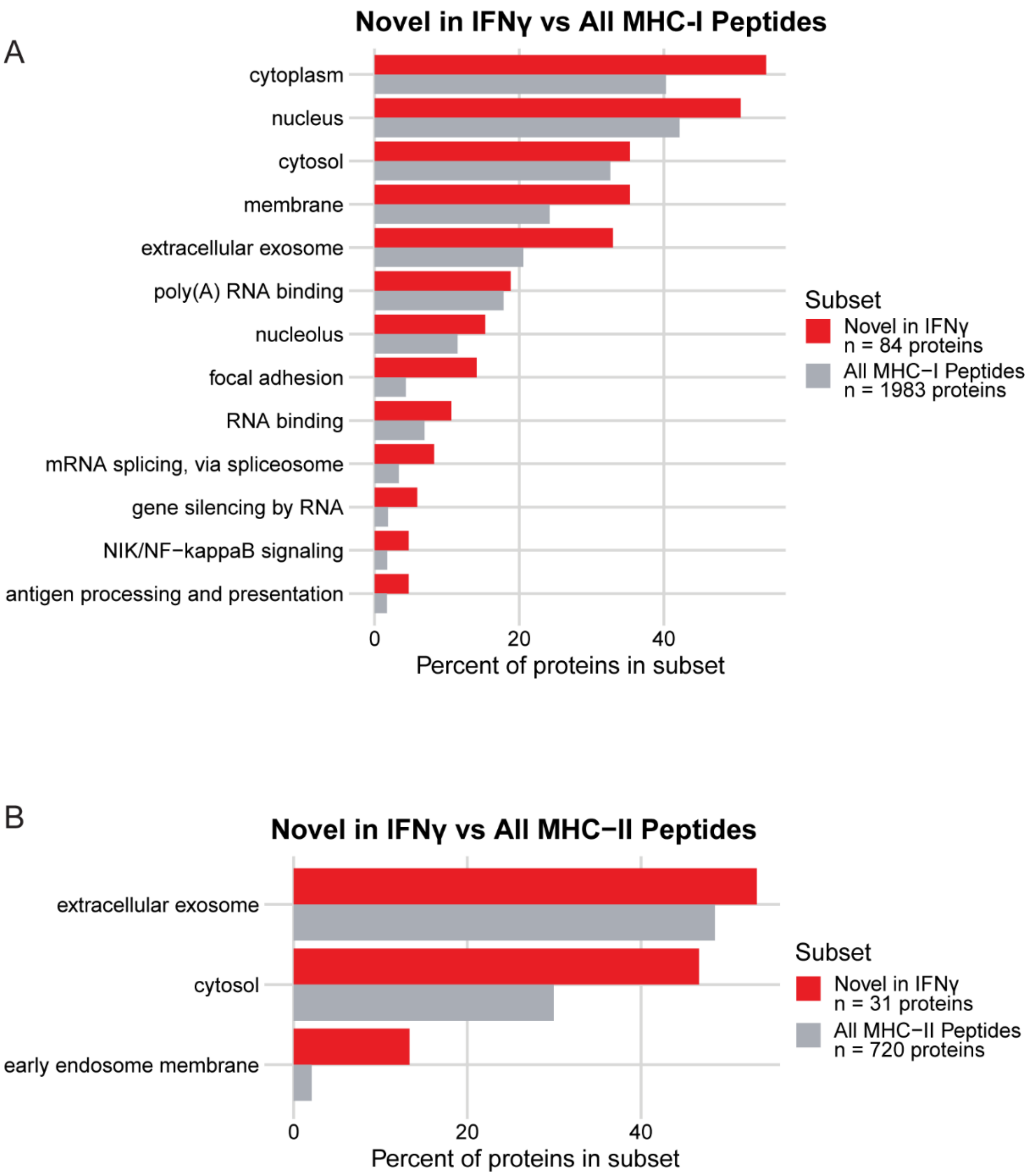

Supplemental Figure 6. GO term analysis of novel pMHC after IFN-γ exposure. (**A**) 84 novel pMHC I and (**B**) 31 novel pMHC II were analyzed using DAVID and compared with all identified pMHC I or pMHC II with proteins identified in the whole-cell proteome experiment as the background. No GO terms were significantly different (FDR > 30%).

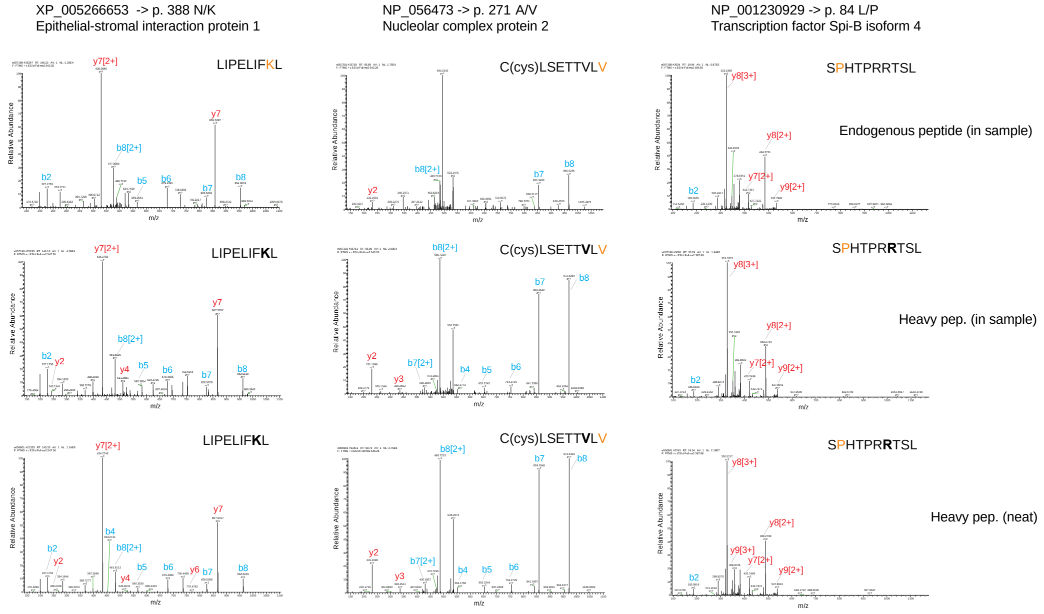

Supplemental Figure 7. MS/MS spectra validation of three differentially expressed SNP-peptides displayed on MHC-I when comparing the IFN-γ vs control state. Amino acid variants indicated in red; isotopically labeled amino acid

1.2 Supplementary Tables

Supplementary Table 1. Proteome-level identifications and multiplexed quantifications
See file “Supplemental_Table1_protein_abundances_TMT_control_IFN_gamma.xls”

Supplementary Table 2. Quantified pMHCI and pMHCII
See file “Supplemental_Table2_IFNg_Control_MHC1_MHC2_quant.xlsx”

Supplementary Table 3. Epitope output from PLAtEAU for pMHCI and pMHCII
See file “Supplemental_Table3_PLAtEAU_MHC1_MHC2.xlsx”

Supplementary Table 4. pMHC changes compared to underlying proteome
See file “Supplementary_Table_4_pMHC_categories.xlsx”
